# Scopoletin Inhibits Rat Aldose Reductase Activity and Cataractogenesis in Galactose-Fed Rats

**DOI:** 10.1155/2013/787138

**Published:** 2013-09-11

**Authors:** Junghyun Kim, Chan-Sik Kim, Yun Mi Lee, Eunjin Sohn, Kyuhyung Jo, So Dam Shin, Jin Sook Kim

**Affiliations:** Korean Medicine Based Herbal Drug Development Group, Herbal Medicine Research Division, Korea Institute of Oriental Medicine (KIOM), 1672 Yuseongdaero, Yuseong-gu, Daejeon 305-811, Republic of Korea

## Abstract

Cataracts are a major cause of human blindness. Aldose reductase (AR) is an important rate-limiting enzyme that contributes to cataract induction in diabetic patients. Scopoletin is the main bioactive constituent of flower buds from *Magnolia fargesii* and is known to inhibit AR activity. To assess scopoletin's ability to mitigate sugar cataract formation *in vivo*, we studied its effects in a rat model of dietary galactose-induced sugar cataracts. Galactose-fed rats were orally dosed with scopoletin (10 or 50 mg/kg body weight) once a day for 2 weeks. Administering scopoletin delayed the progression of the cataracts that were induced by dietary galactose. Scopoletin also prevented galactose-induced changes in lens morphology, such as lens fiber swelling and membrane rupture. Scopoletin's protective effect against sugar cataracts was mediated by inhibiting both AR activity and oxidative stress. These results suggest that scopoletin is a useful treatment for sugar cataracts.

## 1. Introduction

Cataracts are the leading cause of blindness worldwide. Hyperglycemia and diabetes increase the risk of developing cataracts [1]. Cataractogenesis under diabetic or galactosemic conditions is directly linked to the aldose-reductase- (AR-) catalyzed accumulation of sorbitol or galactitol from glucose or galactose, respectively. Accumulating excess sorbitol or galactitol initiates osmotic stress, altering lens cell permeability and redox homeostasis, as well as decreasing ATPase activity and crystallin synthesis [2, 3]. 

Although cataracts can be successfully treated with surgery, it remains important to find nonsurgical treatments for this condition. Synthetic AR inhibitors (ARIs) have been studied to treat diabetic cataracts. The use of traditional medicines, which are mainly derived from plant sources, has remained critical for managing many chronic diseases [4]. Consuming foods or medicinal plants containing micronutrients with potential anti-AR activities may protect against cataracts [5–9]. 

**Figure 1:**
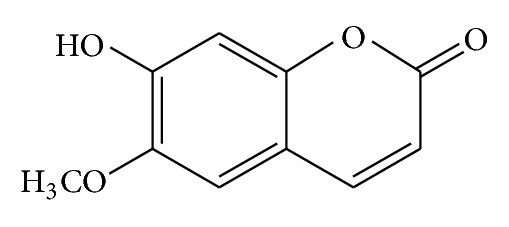
Chemical structure of scopoletin.

Scopoletin (Figure 1) is one of the major coumarin constituents of the flower buds of *Magnolia fargesii*; this plant has been used to treat various inflammatory diseases as a component of traditional Chinese medicines [10]. Several coumarins reportedly block angiogenesis by inhibiting endothelial cell growth [11, 12]. Of the substances found in this plant extract, scopoletin was chosen for study because it possesses a wide range of biological effects, including anti-inflammatory, hypouricemic, and antioxidant activities [13–15]. Recently, scopoletin was reported to regulate hyperglycemia and diabetes [16]. In our previous study, scopoletin from the flower buds of *M. Fargesii* inhibited protein glycation, aldose reductase, and cataractogenesis *ex vivo* [17]. However, *in vivo* anticataract activity and the biochemical mechanism of scopoletin have not been understood yet. In this study, we investigated the effect of scopoletin on galactose-induced cataracts and studied the biochemical mechanism of this protection.

## 2. Materials and Methods

### 2.1. Animals and Experimental Design

To elucidate the effect of scopoletin treatment on sugar cataracts *in vivo*, a galactose-fed rat model was used. Scopoletin was purchased from Sigma (St. Louis, MO, USA). Male Sprague-Dawley (SD) rats (*∼*200 g) were randomized into four groups of 10 rats: Group 1 rats received a normal diet; Group 2 rats received 50% galactose diet (50% w/w with normal diet); Group 3 and Group 4 rats were fed the galactose diet and treated orally with scopoletin (10 and 50 mg/kg body weight, resp.) once a day for 2 weeks. All animal procedures were performed in accordance with the ARVO Statement for the Use of Animals in Ophthalmic and Vision Research and approved by the Korea Institute of Oriental Medicine Institutional Animal Care and Use Committee.

### 2.2. Analysis of Cataract Formation and Its Severity Gradation

After two weeks of treatment, the rats eyes were enucleated under deep anesthesia after an intraperitoneal injection of 10 mg/kg zolazepam (Zoletil, Virbac, Carros, France) mixed with 10 mg/kg xylazine hydrochloride (Rumpun, Bayer, Frankfurt, Germany). The lenses were excised from the eyeball under an optical microscope, and the lenses's wet weights were calculated. The lenses were transferred onto 24-well plates with each well each containing 2 mL saline solution and were photographed under an optical microscope with a CCD camera. The severity of the cataracts was evaluated using the following gradation: grade 0, no opacity; grade I, vacuoles present at a part of the cortical equator; grade II, vacuoles present at all parts of the cortical equator; grade III, vacuoles and their confluents spreading from the cortical equator toward the center of the cortex; grade IV, large, interconnected opacities covering the whole cortex. The opaque areas of the lenses were analyzed using an imaging program (ImageJ, NIH, USA). The data are expressed as the percentage of opaque area relative to the entire lens area.

### 2.3. Analysis of Lens Fiber Degeneration

The isolated lenses were fixed in 10% neutralized formalin for 24 h and embedded in paraffin. To analysis the lens fiber degeneration, fiber cells were visualized by labeling their membranes with wheat germ agglutinin. The lens sections were deparaffinized in xylene and rehydrated. The sections were reacted with 2.5 mg/mL rhodamine-conjugated wheat germ agglutinin (Vector Laboratories, CA, USA) for 60 minutes. All specimens were examined with a fluorescence microscope (BX51, Olympus, Tokyo, Japan).

### 2.4. Determination of AR Activity

A 10% lens homogenate was prepared from two to three pooled lenses in a 50 mM phosphate buffer (pH 7.4). The incubation mixture contained 135 mmol/L Na, K-phosphate buffer (pH 7.0), 100 mmol/L lithium sulfate, 0.03 mmol/L NADPH, 0.04 mmol/L dL-glyceraldehyde, and 150 *μ*L of lens homogenate, in a total volume of 1.0 mL. The reaction was initiated by adding NADPH at 37°C and stopped by adding 0.3 mL of 0.5 N hydrochloric acid. Subsequently, 1 mL 6 N NaOH containing 10 mmol/L imidazole was added, and the mixture was incubated at 60°C for 10 min to convert NADP into a fluorescent product. The fluorescence was measured at room temperature with a spectrofluorophotometer (Ex/Em = 360 nm/460 nm; Synergy HT, Bio-Tek, VT, USA). All measurements were performed in triplicate. 

### 2.5. Lens Polyol Levels

The lens galactitol was measured as previously reported [18]. Briefly, each lens was homogenized in a ground glass homogenizer, and an aliquot of the homogenate was removed for colorimetric protein quantification using a DC Protein Assay (Bio-Rad Laboratories, CA, USA) with bovine serum albumin (BSA) protein standards. Seventy-five microliters of 10% trichloroacetic acid (TCA) was added to 125 *μ*L centrifuged lens homogenate; the mixture was centrifuged at 12,000 rpm for 5 min. To 15 *μ*L protein-free supernatant, we added 50 *μ*L 1 N HCl and 250 *μ*L 25 nM NaIO_4_. The mixture was incubated for 30 min at 37°C. Afterward, 50 *μ*L 1.4 N NaOH and 50 *μ*L 10% ZnSO_4_ were added. The mixture was allowed to stand for a few minutes after vortexing before adding 500 *μ*L 2 M ammonium acetate containing 20 mM pentanedione. The methyltoluidine (absorbance maximum 415 nm) content was measured in the supernatant after incubation for 1 h at 37°C. The polyol standards were treated in the same manner, and the galactitol in the lens homogenate samples was completely recovered.

### 2.6. Glutathione Levels

Each lens was homogenized in a ground glass homogenizer and the insoluble proteins were removed by centrifugation at 4°C. The remaining cell supernatants were deproteinized with equal volumes of 20% TCA, and the glutathione (GSH) levels in the deproteinized supernatant were measured at 412 nm using the DTNB (5-5′-dithiobis[2-nitrobenzoic acid]) method [19].

### 2.7. Immunofluorescence Staining

Lens sections were deparaffinized and hydrated by sequential immersions in xylene and graded alcohol solutions. The slides were placed in 10 mM sodium citrate buffer (pH 6.0) and autoclaved at 121°C for 10 min. Sections were then blocked with normal serum obtained from the same species with a secondary antibody developed to block nonspecific staining. The sections were first labeled with mouse anti-AR antibody (1 : 250, Santa Cruz Biotechnology, CA, USA) overnight at 4°C. After washing, the sections were labeled with fluorescein-isothiocyanate-(FITC-) conjugated goat anti-mouse IgG (1 : 1000, Santa Cruz Biotechnology, CA, USA) for 1 h at room temperature. Finally, the slides were analyzed using fluorescence microscopy (BX51, Olympus). The negative controls for immunostaining were run by incubating the sections with nonimmune serum instead of the primary antibody.

### 2.8. Statistical Analysis

The results were statistically evaluated using a one-way analysis of variance (ANOVA) followed by Tukey's multiple comparison test using GraphPad Prism 5.0 software (GraphPad, CA, USA).

## 3. Results

### 3.1. Cataract Formation Analysis

**Figure 2:**
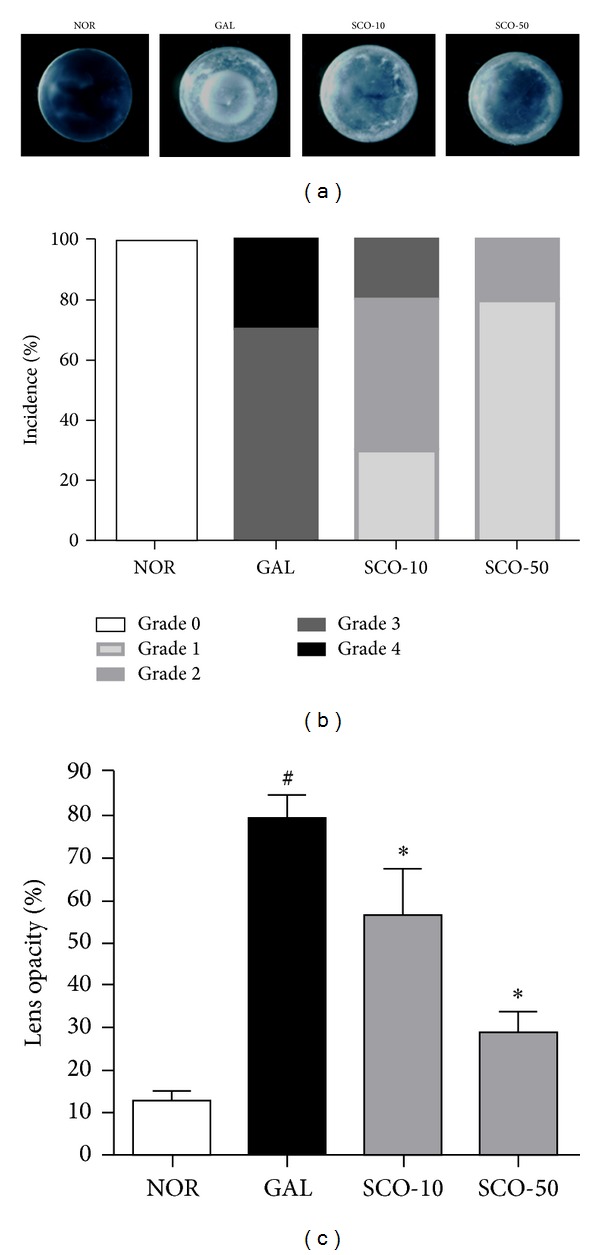
Lens opacity. (a) Representative images of the lenses in each group. (b) Cataract grading. The cataracts were assessed on a scale of 0–4. (c) Analyses of lens opacities. The opacities were analyzed for each lens from the normal rats (NOR), the vehicle-treated galactose-fed rats (GAL), the galactose-fed rats treated with scopoletin at concentration 10 mg/kg (SCO-10), and the galactose-fed rats treated with scopoletin at concentration 50 mg/kg (SCO-50). All data are expressed as the means ± SE, *n* = 10. ^#^ *P* < 0.01 versus normal control rats, *P < 0.01 versus vehicle-treated galactose-fed rats.

During the cataract analysis, all of the animals fed on the galactose diet developed mature cataracts after two weeks (70% were in grade III and 30% were in grade IV, Figures 2(a) and 2(b)). The scopoletin treatment delayed the onset of galactose-induced cataracts in a dose-dependent manner. The highest dose of scopoletin treatment (50 mg/kg/day) delayed the onset of cataracts (80% were in grade I and 20% were in grade II, Figures 2(a) and 2(b)). When analyzing the lenses' opacification, the mean opaque area of the lenses was increased 8-fold in the galactose-fed rats relative to the normal rats; the opacity was suppressed by the scopoletin treatment in a dose-dependent manner (Figure 2(c), *P* < 0.01). Therefore, scopoletin slowed the development of galactose-induced cataracts.

### 3.2. Lens Fiber Cell Degeneration

**Figure 3:**
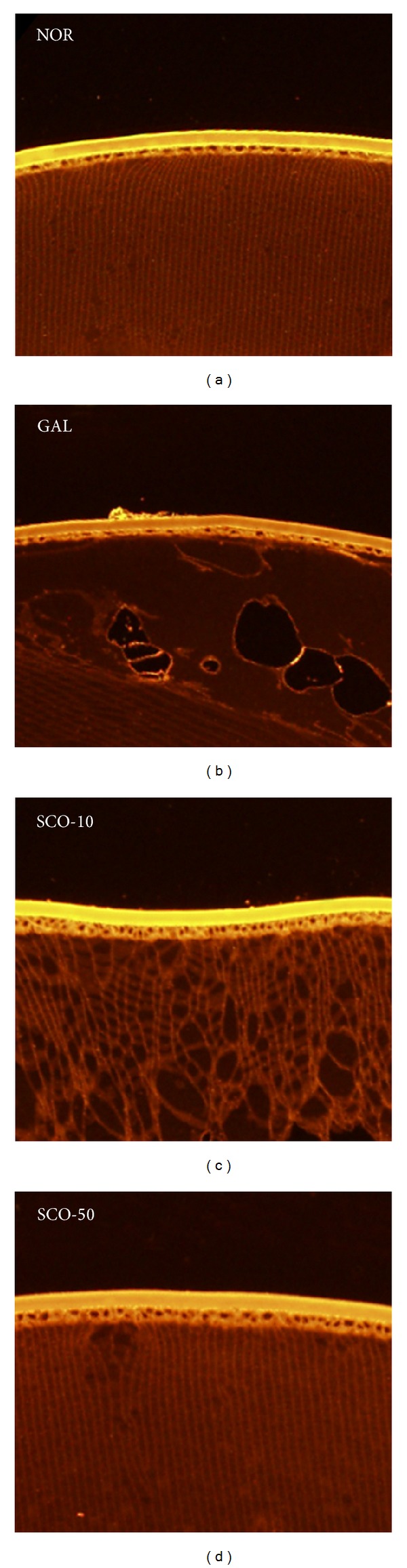
Lens fiber changes. The lens sections from the normal rats (NOR), the vehicle-treated galactose-fed rats (GAL), the galactose-fed rats treated with scopoletin at concentration 10 mg/kg (SCO-10), and the galactose-fed rats treated with scopoletin at concentration 50 mg/kg (SCO-50) were labeled with rhodamine-conjugated wheat germ agglutinin. Fiber cell liquefaction, swelling, and membrane rupture were observed in galactosemic cataractous lens.

In Figure 3, the membrane-labeled lens section illustrates the histological findings after the two weeks of study. No significant alterations in the cuboidal epithelium or lens fiber were observed in the lenses of the control rats. The lenses of galactose-fed rats were swollen, degenerated, vacuolated, and liquefied with degenerated lens fibers. Although the lenticular damage was severe, no corneal or retinal damage was observed. However, this histological change in the lens fibers of galactose-fed rats was prevented in a dose-dependent manner after scopoletin treatment.

### 3.3. Polyol Pathway in Lens

**Figure 4:**
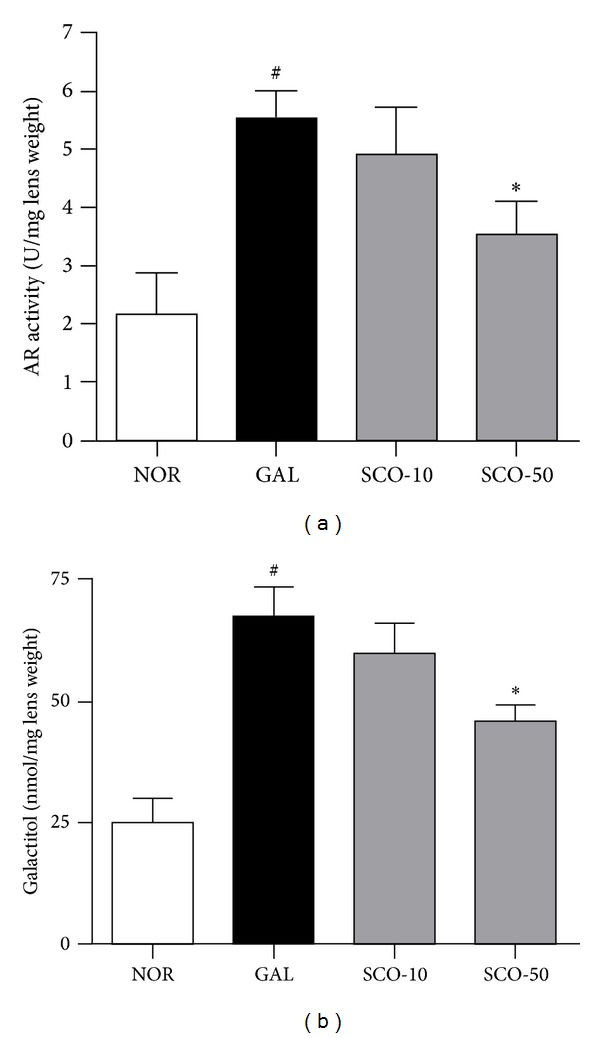
Polyol pathway. (a) Aldose reductase (AR) activity, (b) galactitol levels in lenses from the normal rats (NOR), the vehicle-treated galactose-fed rats (GAL), the galactose-fed rats treated with scopoletin at concentration 10 mg/kg (SCO-10), and the galactose-fed rats treated with scopoletin at concentration 50 mg/kg (SCO-50). All data are expressed as the means ± SE, *n* = 10. ^#^ *P* < 0.01 versus normal control rats, *P < 0.01 versus vehicle-treated galactose-fed rats.

The galactose-fed rats were killed after two weeks; some rats were progressing toward grade 3 cataracts, and extensive protection by scopoletin was observed. AR is a key enzyme in the polyol pathway; its activity was significantly elevated in the galactose-fed rats. The AR activity in the lenses from the scopoletin-treated animals was decreased (Figure 4(a)), agreeing with our observations during our *in vitro* studies [17]. In addition, the galactitol levels in the galactose-fed rats increased relative to the control rats (Figure 4(b)) as expected because the polyol pathway was activated. However, administering scopoletin resulted in less galactose-induced lenticular galactitol accumulation. 

### 3.4. AR Protein Expression in Lens

**Figure 5:**
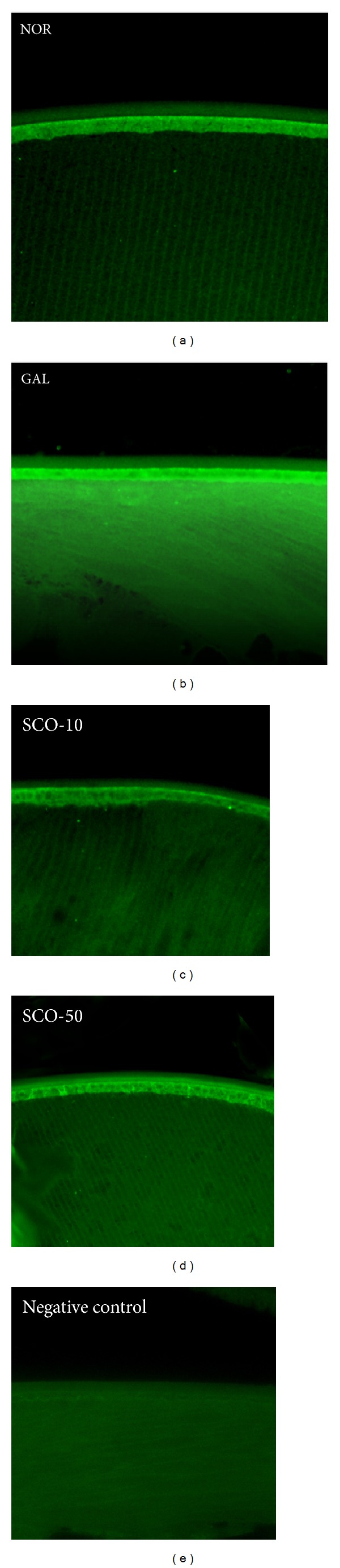
Immunofluorescence stained AR. Representative immunostained AR in lenses from the normal rats (NOR), the vehicle-treated galactose-fed rats (GAL), the galactose-fed rats treated with scopoletin at concentration 10 mg/kg (SCO-10), and the galactose-fed rats treated with scopoletin at concentration 50 mg/kg (SCO-50). AR was strongly immunoreactive in the cytoplasm of the lens epithelial cells and lens cortical fibers. The negative control section was incubated with nonimmune mouse IgG and remained unstained.

In vehicle-treated galactose-fed rats, immunoreactive straining for AR increased in the cytoplasm of lens epithelial cells and extended into the deeper cortical fibers. However, the scopoletin treatment prevented AR expression in the lens epithelial cells and inhibited the extension of AR beneath the epithelial region (Figure 5).

### 3.5. GSH Levels in Lens

**Figure 6:**
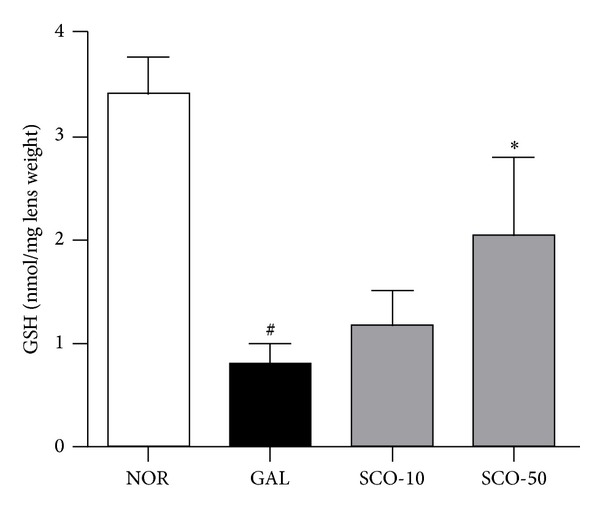
Glutathione (GSH) alteration. GSH was measured in the lenses from the normal rats (NOR), the vehicle-treated galactose-fed rats (GAL), the galactose-fed rats treated with scopoletin at concentration 10 mg/kg (SCO-10), and the galactose-fed rats treated with scopoletin at concentration 50 mg/kg (SCO-50). All data are expressed as the means ± SE, *n* = 10. ^#^ *P* < 0.01 versus normal control rats, *P < 0.01 versus vehicle-treated galactose-fed rats.

The GSH status after treatment indicated that the rats fed with galactose displayed lower GSH levels in their lenses relative to the control. The scopoletin treatment given with the dietary galactose prevented decreases in GSH levels in the lenses (Figure 6).

## 4. Discussion

In this study, we investigated the protective effects of scopoletin against cataractogenesis in galactose-fed rats. The scopoletin treatment delayed the progression and reduced the extent of cataract formation. Currently, the only treatment for cataracts is surgery. It has been estimated that a 10-year delay in the onset and progression of a cataract could reduce the need for cataract surgery by 50% [20].

Galactosemic and diabetic cataractogenesis in experimental animals and humans might be primarily due to the increased formation of polyols from the reduced aldose sugars produced by aldose reductase and nicotinamide adenine dinucleotide phosphate (NADPH) [21]. Polyols may accumulate in the lens fiber cells, causing increased cell hydration, membrane stretching, and dysfunction. Galactose-fed rats are a popular model used to examine the role of the AR pathway in diabetic complications. In addition, the galactose-induced cataracts develop within a week of feeding; this model has been used extensively to study the morphological and biochemical changes during cataractogenesis. Galactitol is a metabolite of galactose by AR that can accumulate in the lens. Because the cellular lens membranes are impermeable to galactitol, hyperosmotic cell swelling occurs, causing light scattering and diminished lens transparency [22–24]. In this study, scopoletin inhibited the lenticular AR activity and the accumulation of galactitol in galactose-fed rats. This AR inhibition corresponded to the anticataractogenic activity. 

ARIs, such as sorbinil, prevented sugar cataractogenesis in experimental animals [25, 26]. Among the ARI, only sorbinil has reached advanced clinical trials in cataract prevention programs. However, due to the manifestation of skin rashes, the trial was discontinued [27]. Although several previous studies demonstrated that ARIs inhibited sugar cataracts by inhibiting AR, no single agent has been proven clinically effective during the treatment of sugar cataracts. Many naturally occurring compounds have strong AR inhibitory activity *in vitro* [28]. Recently, scopoletin demonstrated effective AR inhibitory activity [17, 29]. Coumarins are bicyclic phenolic compounds that harbor a lactone moiety; this functionality might participate in AR inhibition by hydrogen bonding with the TYR48, HIS110, and TRP111 residues in AR [30]. Based on these results, preventing sugar cataracts with scopoletin is partially related to AR inhibition and galactitol accumulation in the lens.

In our previous study, scopoletin had an excellent inhibitory activity on AR, displaying an IC_50_ value of 4.32 *μ*g/mL [17]. Liu et al. reported that the peak plasma scopoletin concentrations (Cmax) were 0.51, 0.68, and 1.49 *μ*g/mL and reached approximately 40 minutes after administering 50, 100, and 250 mg/kg scopoletin in rabbits, respectively [31]. The peak plasma concentration of scopoletin was 8.2 *μ*g/mL after administering 50 mg/kg scopoletin in rats [32]. Because scopoletin was highly lipophilic, it absorbed effectively after oral administration and spread widely to different tissues [33]. Based on its previously reported pharmacokinetics and our *in vitro* results, we chose 10 and 50 mg/kg doses of scopoletin to evaluate its anti-AR activity in rats. We found that the effective dose of scopoletin was 50 mg/kd, agreeing with the *in vitro* result.

The enzymatic distribution of AR activity was supported by AR's localized immunofluorescence. In human and rat lenses, AR is primarily localized in the epithelial and superficial cortical fiber cells [34]. In galactose-fed rats, the enhanced immunoreactive staining of AR was observed in the epithelial cells and the cortex region. This staining decreased, progressing from the superficial region to the deeper cortex. Scopoletin inhibited the extension of AR beneath the epithelial region. Therefore, the decrease in AR activity observed in the scopoletin-treated rats was caused by the reduced amount of AR protein.

Although the prevention of sugar-induced cataractogenesis by ARIs appears to be caused by AR inhibition, the osmotic hypothesis might not fully explain diabetic cataracts in human subjects because, even during severe hyperglycemia, the examined tissues, including the lens, did not have sorbitol levels >2 mM [35]. Antioxidants effectively slow sugar cataract formation. Butylated hydroxytoluene is a well-known synthetic phenolic antioxidant that slows cataract formation in rat lenses cultured under high-glucose conditions, although the sorbitol and fructose levels in the lenses remains elevated [36]. Consequently, Wolff and Crabbe suggested that the ARIs protected against sugar cataracts due to the antioxidant nature of these inhibitors [37]. Scopoletin has demonstrated benefits for oxidative injury as an antioxidant [38, 39]. In this study, scopoletin preserved the lenticular GSH content. Therefore, one of the possible mechanisms for scopoletin during sugar cataract development may involve the protection of the lens cell membrane from oxidative damage.

In summary, this study reveals that scopoletin may exert beneficial/protective effects during the sugar cataract development. Scopoletin inhibits the AR activity, polyol accumulation, and reduction of the GSH levels. We suggest that the scopoletin may be particularly useful in treating sugar cataracts.
